# Assessing risk indicators of intimate partner femicide considering victim's coping strategies to violence: A dynamic multilevel linear mixed model based on genetic algorithms

**DOI:** 10.1016/j.heliyon.2024.e37827

**Published:** 2024-09-13

**Authors:** Esperanza Garcia-Vergara, Francisco Fernández-Navarro, David Becerra-Alonso, Nerea Almeda

**Affiliations:** a Department of Quantitative Methods, Universidad Loyola Andalucía, Seville, 41704, Andalusia, Spain; b Department of Computer Languages and Computer Science, Universidad de Málaga, Málaga, 29071, Andalusia, Spain; c Department of Psychology, Universidad Loyola Andalucia, Seville, 41704, Andalusia, Spain

**Keywords:** Intimate partner violence, Intimate partner homicide, Femicide, Domestic violence, Risk factors

## Abstract

Intimate partner femicide (IPF) is a grave social and health concern affecting women worldwide, with approximately 30,000 deaths annually at the hands of their current or former intimate partners. Previous studies have focused on identifying risk factors for IPF and developing risk assessment tools to identify high-risk cases. However, an important aspect that has been overlooked in these studies is victims' coping strategies in response to intimate partner violence. Understanding victims' coping strategies can provide valuable insights into how they deal with the abuse and can inform the development of effective interventions and prevention strategies. This study aims to address this gap by developing a multilevel linear mixed model (LMM) to analyze the impact of engagement and disengagement coping on the likelihood of IPF and identify common and specific IPF risk indicators for these coping strategies. A total of 491 Spanish cases of violence against women by current or former intimate partners were analyzed from penal sentences issued by Spanish provincial and supreme courts from 2019 to 2022. The LMM model obtained from the study has competitive performance in identifying IPF and non-IPF cases, including risk indicators of prior history of injuries, history of sexual aggression, frequency and escalation of violence, physical violence, place of crime, lonely place of crime, and community presence. Victims with engagement coping and disengagement coping share some risk indicators, while others belong to just one category. Overall, the results suggest that victims with disengagement coping are more predisposed to suffer IPF than victims with engagement coping. This evidenced-based knowledge emphasizes the significance of considering coping strategies in predicting and preventing IPF, with further implications discussed at the end of this paper.

## 1 Introduction

The United Nations defines violence against women as “*any act of gender-based violence that results in, or is likely to result in, physical, sexual, or mental harm or suffering to women, including threats of such acts, coercion or arbitrary deprivation of liberty, whether occurring in public or in private life*” [1]. Intimate partner violence (IPV) is the most common form of violence against women, affecting 641 million female victims worldwide [2]. IPV refers to both male and female perpetration and victimization, but in this study, this term is used to refer exclusively to partner violence by men against women [3], [4]. In the most severe cases, IPV can result in women's deaths, which are referred to as intimate partner femicide (IPF) [5], [6], [7]. Every year, around 30,000 women are killed worldwide by their current or former intimate partners, making it the leading cause of violent death for females [8], [9], [10]. According to several studies, the rates of women who suffer homicide at the hands of their intimate partner is significantly higher than that of men [11], [12], [6]. The proportion of women murdered by their intimate partners is six times higher than that of men [9]. This highlights the importance of studying IPF from a gender perspective, separating it from the male victimization phenomenon.

Intimate partner femicide (IPF) is a complex phenomenon influenced by a range of factors related to the victim and offender. The meta-analysis by Spencer [13] suggest that the strongest risk factors for IPF include victim pregnancy, substance abuse, lack of secondary education, separation or divorce, and presence of stepchildren. However, most of the previous studies have primarily focused on individual-level risk factors, overlooking the potential role of environmental and situational factors in IPF. The systematic review by Garcia-Vergara et al. [14] highlights the importance of taking into account the environment and its contextual elements in the study of IPF. Some research has focused exclusively on the neighborhood's geographic setting and situational characteristics where violence against women occurs. Notably, the studies by Edwards [15], Gallup-Black [16], and Gillespie and Reckdenwald [17] suggest that geographic location influences IPF perpetration. Their findings reveal a significant discrepancy in IPF rates between rural and urban areas, being higher in rural areas. Additionally, Beyer et al. [18] and Benson et al. [19] have found a link between neighborhood and intimate partner violence. They found that a reduced cohesion between neighbors elevates the risk of violence against a partner. By contrast, supportive social networks and solid social ties reduce the risk.

Not only the context is relevant in the consideration of IPF, but also the coping strategies. Victims employ various coping mechanisms to manage the physical and psychological effects of violence [20], [21], [22], [23], [24], [25]. Coping, as defined by Lazarus and Folkman [26], refers to cognitive and behavioral efforts to manage specific stressors and demands that are evaluated by the individual as exceeding or overwhelming their resources. In the context of IPV, coping refers to attempts by women to reduce the overall psychological stress resulting from abuse [27], [24]. Problem-focused coping and emotion-focused coping are the most commonly used coping strategies in this area [28]. Problem-focused coping refers to actions focused on generating changes in the source of stress, which concerns the victimization. Emotion-focused coping constitutes emotional reactions that help to manage that stressful situation [29], [30], [26], [31]. Engagement and disengagement coping are also common referred strategies. Engagement coping involves proactive actions to manage the violence and its effects, such as help-seeking, while disengagement coping refers to the opposite involving problem avoidance and self-criticism [32], [33].

Numerous studies have investigated coping strategies used by women who are victims of IPV. Six major research lines can be identified in this regard. The first line focuses on identifying the range of coping responses adopted by women who have experienced IPV [34], [35], [36], [37]. The second line aims to explore coping strategies in specific victim groups, such as elderly women [38], [39], immigrant women [40], [41], pregnant women [42], [43], and women with mental health problems [44], [45]. The third line analyzes the impact of the environment on coping behaviors adopted by victims, including those in rural areas [46], [47]. The fourth line explores effective and ineffective coping strategies in preventing and stopping IPV [48], [49]. The fifth line examines the relationship between coping strategies and help-seeking, determining what types of resources victims access based on the coping strategies developed [25]. The sixth line analyzes the effect of coping strategies on IPV. Disengagement coping is associated with a higher prevalence of victimization and future revictimization risk, while engagement coping strategies can protect women from future IPV by effectively using personal resources to reduce distress.

Therefore, engagement coping protects women from violence by enabling them to use resources effectively [44], [32]. Although coping is a critical element in predicting and preventing IPF, little is known about the mediation effect of victims' coping on IPF. To date, no studies have explored this relationship. This study aims to contribute to the literature on IPF by examining the impact of engagement and disengagement coping on the probability of IPF occurrence. Moreover, the study seeks to identify risk indicators of IPF for each coping strategy (as femicide has been studied differently for each coping strategy), including environmental factors, and takes into account the stratified nature of the phenomenon, which has not been considered in prior research. To achieve these objectives, we propose ad-hoc a novel multilevel linear mixed model. The use of multilevel modeling is a powerful approach to address the challenge of modeling both common and specific factors associated with IPF risk. This method allows for the incorporation of random effects for different coping strategies, which can help to capture how the relationship between coping strategies and IPF risk may vary across individuals while accounting for the common factors that may be associated with IPF risk across all individuals. The proposed methodology strategy leverages a heuristic approach, which operates from data to theory, allowing us to identify potential novel interactions. Although this method may require more exploration time, it offers the advantage of investigating interactions not covered in the existing literature.

Additionally, the study will identify common and specific risk factors for IPF based on these coping strategies, utilizing secondary data obtained from the Vlex legal database.^1^ By including both engagement and disengagement coping styles in the analysis, this study will provide a more comprehensive understanding of the relationship between coping strategies and IPF. Furthermore, utilizing secondary data from the Vlex legal database offers a comprehensive exploration of the risk factors associated with IPF, providing potential indicators that would not otherwise be possible to know due to the death of the women. By using Vlex data, we can obtain a representative sample of victim groups with both engagement and disengagement coping strategies for both IPF and non-IPF. This research could offer valuable insights into the effectiveness of interventions and services in preventing lethal violence.

## 2 Methodology

### 2.1 Database of the study

#### 2.1.1 Data acquisition

To obtain data for the study, the cases of violence against women by current or former intimate partners were extracted from the Vlex legal database. The search was conducted on February 13th, 2022 and updated on January 3rd, 2023, following specific search criteria that included crime, judicial resolution, jurisdiction, jurisdictional organ, time, and place. These criteria were applied to ensure that the selected cases met the study's requirements and were relevant to the research questions.

The first criterion was based on the type of crime, which included violence against women by current or former intimate partners' offenses exclusively. To identify such cases, the Spanish term ‘violencia de genero’ (referring to violence against women by current or former intimate partners' crimes) was entered into the database, along with specific articles from the Spanish penal code that refer to these crimes (articles 138, 139, 140, 147, 148, 149, 150, 153, 163, 164, 166, 169, 169, 172, 173, 178, 179, 180, 181, 187, 197, 208, and 209). This search yielded a total of 197,285 cases, which were then narrowed down to 51,342 when combined with the articles from the Spanish penal code.

The second criterion pertains to judicial resolutions, specifically, only final sentences were included, while provisional resolutions were excluded. As a result, the initial pool of cases was reduced from 51,342 to 39,010. The third criterion concerns jurisdiction and is limited to the penal field. This entails selecting only penal sentences, while excluding sentences related to civil, fiscal, social, constitutional, commercial and business, public and administrative, and procedural law. By applying this criterion, the number of cases was reduced from 39,010 to 37,856.

The fourth criterion is related to the jurisdictional organ and involves selecting only the provincial and supreme courts. By applying this criterion, the initial number of 37,856 cases was reduced to 36,537. The fifth criterion pertains to the time period, limiting the cases to recent years between 2019 and 2022. As a result, the 36,537 cases were further reduced to 14,753. The sixth criterion focuses on the place, restricting the selection to only cases that occurred in Spain, and not in other countries. After applying this criterion, the total number of cases was further reduced to 8,481.

Out of the 8,481 cases that were selected through the previous criteria, the following were excluded: (1) sentences that resulted in acquittals, (2) sentences that involved at least one partner who was a minor, (3) sentences related to reciprocal violence between partners, (4) sentences that annulled previous judicial resolutions, (5) sentences without proven facts, and (6) sentences written in co-official languages of Spain.

Thus, the present study selected a final set of 491 cases of violence against women by current or former intimate partners that were condemned in penal sentences by Spanish provincial and supreme courts between 2019 and 2022. Out of the total cases, 330 were categorized as non-IPF, involving crimes against physical and psychological integrity, personal freedom, sexual integrity, and privacy committed by a husband, ex-husband, or partner/ex-partner of a woman. The remaining 161 cases were categorized as IPF, which included homicides and murders.

Finally, Table 1 offers a detailed breakdown of the socio-demographic characteristics of the study's sample. This information is essential for understanding the sample used to develop the model, and it guides our subsequent discussion. It's particularly valuable when comparing victim groups using disengagement and engagement coping strategies, providing insights into how these characteristics can impact the study's outcomes.

**Table 1:** Frequency distribution of sociodemographic characteristics among the women victims in the study sample, including those with disengagement and engagement coping strategies.

	Total (%)	With disengagement coping (%)	With engagement coping (%)
**Age**			
18-24	15.3	67.1	32.9
25-34	24.6	16.3	83.7
35-44	26.4	30.8	69.2
45-54	28.7	66.9	33.1
55-64	2.1	48.1	51.9
65 and older	2.9	58.9	41.1

**Nationality**			
Spanish	66.9	38.8	61.2
Non-spanish	33.1	53.4	46.6

**Marital status**			
Single	36	15.8	84.2
Married	37.7	43.3	56.7
Separated	10.3	24.4	75.6
Divorced	16	16.7	83.3

**Employment status**			
Employed	29.6	31.2	68.8
Unemployed	70.4	77.9	22.1

**Geographic location**			
Rural	51.5	39.1	60.9
Urban	48.5	33.6	66.4

#### 2.1.2 Variables employed in the study

The dependent variable in this study is intimate partner femicide (IPF). To operationalize it, a binary coding scheme was used. Specifically, a value of 1 was assigned to IPF, indicating the presence of crimes against life, while a value of 0 was assigned to non-IPF, indicating the absence of crimes against life in the intimate partner violence against women context.

The study includes thirty-nine independent variables that are related to criminal records, criminal history, characteristics of violence, environment, and situation of the crime. In terms of criminal records, there are two independent variables that refer to past crimes for which the perpetrator was convicted by judicial authorities: (i) general criminal records ($x_{1}$) refers to any criminal records not related to IPV and (ii) specific criminal records of IPV ($x_{2}$) only includes criminal records on IPV. These variables are binary and take values of 0 or 1, indicating the absence or presence of past convictions, respectively.

There are five independent variables that provide additional information on the specific criminal record of IPV, with values of 0 and 1 indicating absence or presence, respectively. These variables are:

• Criminal records of injuries of IPV ($x_{3}$)^2^
• Criminal records of threats of IPV ($x_{4}$)^3^
• Criminal records of constraints of IPV ($x_{5}$)^4^
• Criminal records of habitual violence of IPV ($x_{6}$)^5^
• Criminal records of insults of IPV ($x_{7}$)^6^

Data on sentences related to criminal records of IPV and their duration have been collected as independent variables. The variables related to sentences take values of 0 and 1, corresponding to the absence or presence of the sentence considered, while the time-related variables are numerical. The variables considered are^7^:

• Custodial sentence ($x_{8}$) refers to deprivation of freedom sentences.
• Non-custodial sentence ($x_{9}$) involves sentences without deprivation of freedom.
• Prison sentence ($x_{10}$) and its duration ($x_{16}$).
• Deprivation of the right to possess and carry weapons sentence ($x_{11}$) and its duration ($x_{17}$).
• Prohibition to approximate the victim sentence ($x_{12}$) and its duration ($x_{18}$).
• Prohibition to communicate with the victim sentence ($x_{13}$) and its duration ($x_{19}$).
• Community service sentence ($x_{14}$) and its duration ($x_{20}$).
• Fine sentence ($x_{15}$) and its duration ($x_{21}$).

The criminal history of IPV is another set of independent variables that refers to past crimes that were not registered as criminal records by judicial authorities. These crimes were sentenced at the same time as other recently perpetrated crimes, either because the authorities were not aware of them or because there was insufficient evidence to sentence the offender at that time. The presence or absence of these variables has been recorded using binary values of 0 and 1. The variables included in this category are:

• History of injuries of IPV ($x_{22}$)
• History of threats of IPV ($x_{24}$)
• History of constraints of IPV ($x_{25}$)
• History of habitual violence of IPV ($x_{26}$)
• History of insults of IPV ($x_{30}$)
• History of illegal detention of IPV ($x_{23}$)^8^
• History of sexual aggression of IPV ($x_{27}$)^9^
• History of sexual abuse of IPV ($x_{28}$)
• History of invasion of privacy of IPV ($x_{29}$)^10^

The characteristics of violence are reflected in four independent variables:

• The frequency of violence ($x_{31}$) takes on a value of 0 for low frequency (1 violent incident), 0.5 for medium frequency (2-3 violent incidents), and 1 for high frequency (4 or more violent incidents).
• The escalation of violence ($x_{32}$) assumes a value of 0 when there is not a gradual increase in the frequency and severity of violence over time, and a value of 1 when there is escalation.
• Physical violence ($x_{33}$) refers to assaults that cause physical harm, taking on a value of 0 when it is absent and a value of 1 when it is present.
• Psychological violence ($x_{34}$) involves attacks on mental health, taking on a value of 0 when it does not occur and a value of 1 when it does.

In terms of the environment and situation surrounding the crime, there are five independent variables to consider:

• Place of crime ($x_{35}$) takes a value of 0 for crimes committed in rural areas, and 1 for those in urban areas.
• Time of crime ($x_{36}$) assumes a value of 0 for crimes committed at dawn, 0.25 for morning, 0.50 for afternoon, 0.75 for night, and 1 for various times.
• Situational characteristics of crime ($x_{37}$) refers to whether the crime scene is a public area or a secluded one, with a value of 0 for the former and 1 for the latter.
• Community presence ($x_{38}$) takes a value of 0 when there are no people present at the place and time of the crime, and 1 when there are witnesses or potential interveners.
• Discussion ($x_{39}$) relates to whether there was a verbal altercation between the partners immediately prior to the occurrence of the crime, with a value of 0 indicating the absence of such a discussion, and 1 indicating its presence.

Apart from the dependent and independent variables, the study includes a stratification variable known as victim's coping strategies, which sheds light on the actions taken by the victim in response to violence by their intimate partner. This variable is critical for achieving the research objective of estimating IPF based on specific risk indicators in cases with engagement and disengagement victim's coping strategies. The victim's coping strategies have been classified as engagement and disengagement coping strategies. The former includes help-seeking, denouncing, and leaving the relationship, while the latter includes negation, silence, and conflict avoidance. By analyzing the data based on these coping strategies, the study aims to provide a more nuanced understanding of the dynamics of intimate partner violence and the effectiveness of different coping strategies.

Finally, it is important to stress that several other independent variables were recorded in addition to those described in the previous paragraphs. However, these had to be removed from the study because they had a high rate of missing values, which would have compromised the statistical analysis and the reliability of the results.

### 2.2 Data analysis

This section presents the complete mathematical procedure implemented to explain the IPF variable in terms of previously defined predictor variables. The procedure is divided into five parts: (i) the description of the data preprocessing techniques carried out in the study, (ii) the functional structure of the model and the estimation of its parameters, (iii) the procedure proposed to select a subset of predictor variables optimally,^11^ (iv) the classification rule implemented in the study and (v) the performance validation metrics employed in the study.

#### 2.2.1 Data preprocessing

Data preprocessing is a crucial step in statistical learning that involves cleaning, transforming, and preparing raw data for analysis. The main goal of data preprocessing is to improve the quality and usability of the data, enabling the machine learning model to perform better. This section focuses on three data preprocessing techniques carried out in the study: data scaling, data imputation, and feature interaction expansion.

1. **Data scaling**. The variables included in the study were all linearly scaled 0-1 at the beginning of the procedure. Scaling can improve the interpretability of the model, as it allows for easier comparison of the relative importance of each variable. It can also enhance the performance of distance-based [50] and gradient descent algorithms [51] by mitigating the influence of variables with larger values, leading to more stable and optimal results.
2. **Data imputation**. In data analysis, missing data can significantly impact the quality and accuracy of statistical models. One common approach to handling missing data is data imputation, which involves estimating the missing values based on the observed data. Of all the existing imputation techniques, the implemented one is the moving window median approach which calculates the median of a sliding window of adjacent data points to estimate missing entries. The appropriate window length should balance the accuracy of the imputed values and the statistical properties of the data. In the experiments, the window length was set to 100, and the data imputation procedure was implemented in MATLAB and is available upon request to the authors.
  The number of imputed data for each variable in the study was different. For the criminal records and criminal history variables, the data imputation rate was below 1 percent. In the case of variables related to sentences, discussion, situational characteristics of crime, and frequency of violence, the data imputation rate was moderated. Specifically, between 2 and 3 percent of the data were imputed for the sentences and discussion variables. For the situational characteristic of the crime variable, 4 percent of its data was imputed, while the frequency of violence variable reached an imputation rate of 5 percent.
  The highest data imputation rates were observed in the characteristics of violence and environmental variables (excluding frequency of violence, discussion, and situational characteristics of crime, as stated above). In particular, 10 percent of data was imputed for the time of crime variable. In the case of the escalation of violence variable and physical and psychological violence variables, data imputation rates ranged from 10 to 13 percent. Lastly, the data imputation rate for the place of crime and community presence variables ranged between 14 and 16 percent.
3. **Feature interaction expansion**. In this stage, new features resulting from combining or interacting pairs or higher-order combinations of existing features are created to improve the model's predictive power. Specifically, the original input data (composed of 39 predictor variables, K=39) was extended to include all the possible first-order interaction terms (741 in this study). Thus, the features incorporated in the final model include both the original independent variables (39) and first-order interaction terms (741), resulting in a total of 780 input features, $39+\left(\begin{array}{c} 39\\ 2 \end{array}\right)$.

#### 2.2.2 Functional structure and parameters estimation for the Linear Mixed Model (LMM)

The functional structure of the Linear Mixed Model (LMM) for each group cluster $G_{i}$, 1≤i≤m (taking into account that the problem being analyzed is composed of two clusters, m=2, women with engagement and disengagement coping strategies), is defined by the following equation:

$$y_{i}=P_{i}\beta +Z_{i}b_{i}+\epsilon_{i},$$

where $y_{i}\in {\left\{0,1\right\}}^{n_{i}}$ is the vector with the target outputs (encoded as binary variables), $n_{i}$ the number of patterns associated with the *i*-th cluster (being $N=\sum_{i=1}^{m}n_{i}$ the total number of patterns of the whole problem), $P_{i}\in R^{n_{i}\times p}$ the matrix for the fixed effects (with *p* being the number of variables included in the fixed effects set), $\beta \in R^{p}$ the vector of fixed effects parameters, $Z_{i}\in R^{n_{i}\times z_{i}}$ the input matrix of the random effects associated with the group cluster $G_{i}$, $z_{i}$ the number of variables included in the random effects set of $G_{i}$, $b_{i}\in R^{z_{i}}$ the vector of random effects parameters, and $\epsilon_{i}\in R^{n_{i}}$ the vector of random errors which are assumed to be independent of $P_{i}$, $Z_{i}$ and $b_{i}$. For the sake of clarity, let $y=(y_{1},y_{2},\ldots ,y_{m})\in {\left\{0,1\right\}}^{N}$, $\epsilon =(\epsilon_{1},\epsilon_{2},\ldots ,\epsilon_{m})\in R^{N}$ be N×1 vectors and $P=(P_{1},P_{2},\ldots ,P_{m})\in R^{N\times p}$ the complete design matrix for the fixed effects variables.

The distributional assumptions of the LMM model are:

$$\epsilon_{i}∼N(0,\sigma^{2}I_{n_{i}}),b_{i}∼N(0,\sigma^{2}D),$$

where $D\in R^{q\times q}$ is a symmetric nonnegative definite matrix that it will be estimated using the approach proposed in [52].

The proposed LMM model, which is a regression model, has been used to address a binary classification problem assuming a regression approach 0-1. This means that instead of directly classifying the input data into one of the two classes ($C_{0}$ or $C_{1}$), the LMM model treats the problem as a regression problem and predicts a continuous value between 0 and 1. Then, a threshold value is used to determine the predicted class based on whether the predicted value is above or below the threshold. This approach is commonly used in binary classification problems and has been shown to be effective in various applications.

For all the reasons mentioned above, the initial estimate of the fixed effects coefficients is obtained through the ordinary least squares problem, which results in the following estimation of parameters:

$$\overset{ˆ}{\beta }={\left(P^{T}P\right)}^{-1}P^{T}y.$$

Using the initial estimate, it is important to remember that $y_{i}-P_{i}\beta =Z_{i}b_{i}+\epsilon_{i}$. Next, the random effect for each group cluster $G_{i}$, where 1≤i≤m, should be estimated using the least squares method as:

$${\overset{ˆ}{b}}_{i}={\left(Z_{i}^{T}Z_{i}\right)}^{-1}Z_{i}^{T}(y-P_{i}\overset{ˆ}{\beta }).{\overset{ˆ}{\epsilon }}_{i}=(y-P_{i}\overset{ˆ}{\beta })-Z_{i}{\overset{ˆ}{b}}_{i}.$$

Using these estimates, the subsequent estimators for $\sigma^{2}$ and **D** are calculated [52]:

$${\overset{ˆ}{\sigma }}^{2}=\frac{\sum_{i=1}^{m}{\overset{ˆ}{\epsilon }}_{i}^{T}{\overset{ˆ}{\epsilon }}_{i}}{N-\sum_{i=1}^{m}z_{i}}.\overset{ˆ}{D}=\frac{\sum_{i=1}^{m}({\overset{ˆ}{b}}_{i}{\overset{ˆ}{b}}_{i}^{T})}{m{\overset{ˆ}{\sigma }}^{2}}-\frac{\sum_{i=1}^{m}{\left(Z_{i}^{T}Z_{i}\right)}^{-1}}{m}.$$

With the computation of $\overset{ˆ}{D}$, the fixed effects are re-estimated using weighted least squares as follows:

$$\overset{ˆ}{\beta }={\left(P^{T}WP\right)}^{-1}P^{T}Wy,$$

where $W=\mathrm{diag}\{{\left(Z_{1}\overset{ˆ}{D}Z_{1}^{T}+I_{n_{1}}\right)}^{-1},\ldots ,{\left(Z_{m}\overset{ˆ}{D}Z_{m}^{T}+I_{n_{m}}\right)}^{-1}\}$. Finally, an iterative process is employed by recomputing ${\overset{ˆ}{b}}_{i},{\overset{ˆ}{\epsilon }}_{i},{\overset{ˆ}{\sigma }}^{2}$ and $\overset{ˆ}{D}$ using the new estimates of $\overset{ˆ}{\beta }$ until convergence. This process is continued until the convergence is achieved. This procedure for estimating the fixed and random effects parameters has already been proposed in [52].

It is important to highlight that the statistical model proposed in the study has been programmed by the research team involved in the study and is available upon request to the authors.

#### 2.2.3 Variable selection

The matrices defining the fixed and random effects, $P,Z_{1},\ldots ,Z_{m}$, are dynamically determined via a Genetic Algorithm (GA) expressly implemented ad-hoc (in MATLAB) for the study. Those matrices are all sub-samples of the expanded training dataset, and consequently, the number of rows depends on the sample size of the clusters being analyzed, $n_{i}$, 1≤i≤m, whereas the columns on the attributes included (model selection) in each random effect (per cluster) and the fixed effects. Thus, the GA has been conducted m+1 times (3 in the study), the first one to select the variables included as fixed effects (*p*), and the other two to identify the subset of regressors to be included in the random effects group clusters ($z_{1},\ldots ,z_{m}$).

In this way, the proposed GA heuristically explored the search space to find a reduced set of regressors with high explanatory power to be included in the LMM within the fixed effects part and the random effects sets. Hence, the GA considers a population of candidate solutions representing different subsets of predictors (independent variables and first-order interaction terms), which evolve towards better classification models. The algorithm begins from a population of randomly generated subsets of predictors. An array of binary values represents each individual. Then, a fitness score is assigned to every individual. The fitness is composed of two objectives: the complexity of the model and its performance. After that, the mutation and crossover operators modify the population to explore the search space, and this procedure is repeated during *G* generations (150 in the experiments). The principal characteristics of the GA are described in continuation.

• **Encoding and initialization of the population**. The encoding for the different solutions is a binary array with 780 components, representing each element if the corresponding independent variable or interaction term is included or not in the final model (Integer Genetic Algorithm, IGA). Hence, each individual could be defined as:
  $$s\in {\left\{0,1\right\}}^{K+\left(\begin{array}{c} K\\ 2 \end{array}\right)},$$
  where $s_{i}=1$ if the *i*-th variable is included in the LMM, and 0 otherwise, $i=1,\ldots ,K+\left(\begin{array}{c} K\\ 2 \end{array}\right)$, (K=39). The initial population of candidates is generated randomly, and the parameter associated to the number of individuals in the experiments was set to 60.
• **Fitness function**. The fitness function implemented is based on the Bayesian Information Criterion, which weights the model performance with its complexity (measured in terms of the number of variables included in the model):
  $$\underset{s}{\min}\;f(s)=l\ln(e_{s}^{2})+\lambda h\ln(l)s.t.\;s\in {\left\{0,1\right\}}^{K+\left(\begin{array}{c} K\\ 2 \end{array}\right)},$$
  where *l* is the sample size of the problem analyzed (out of the m+1 total executions of the GA), $l\in \{N,n_{1},\ldots ,n_{m}\}$, *h* the number of the variables included in the model, $h\in \{p,z_{1},\ldots ,z_{m}\}$, λ>0 defines the importance of the second objective with respect to first one, and $e_{s}^{2}$ the error variance of the individual evaluated (**s**). The first part estimates the model's accuracy, whereas the second one its complexity. In the experiments, the parameter *λ* was set to 4.
• **Crossover and mutation**. The crossover operator implemented was the single-point crossover with a random pairing process that assigns an equal probability to each candidate. This crossover operator produces new offspring from the individuals chosen to be combined. The mutation operator randomly changes the value in a cell of the candidate designated to be mutated. The mutation rate in the experimentation is set to 2%.

#### 2.2.4 Classification rule

When dealing with binary classification problems, a common approach is to use statistical learning models that treat the problem as a 0-1 regression problem. In this research, the proposed LMM model is trained on a labeled dataset where the target variable has either 0 or 1, corresponding to the two possible classes. The model then learns a regression function that predicts the positive class probability (i.e., $C_{1}$) for each input instance.

Under this formulation, defining a decision threshold to determine the predicted class knowing the estimated probability is crucial, which is the output of the LMM model. If the predicted probability is above the threshold, the pattern is classified as belonging to the positive class. Otherwise, it is classified as belonging to the negative class (i.e., $C_{0}$).

The choice of the threshold depends on the desired trade-off between precision and recall and can be adjusted to optimize performance on the specific task at hand. For simplicity, we have fixed in the experiments the threshold to 0.5, although it could be modified to promote better classification of one class to the detriment of the accuracy of the other class.

#### 2.2.5 Performance validation metrics

To evaluate the performance of the LMM model, several metrics have been used for regression (Root Mean Squared Error and R-Squared) and classification (Correctly Classified Rate).

• Root Mean Squared Error (RMSE) measures the deviation of the predicted values from the actual values in the model. It provides information about the model's accuracy, based on the distance of the data from the regression line.
• R-Squared (R^2^) indicates the proportion of total outcome variance that can be explained by the predictors or explanatory variables included in the model.
• Correctly Classified Rate (CCR) is a measure of the model's ability to correctly predict or classify cases. In this study the global CCR of the LMM model is taken referring to the correct detection rate ofIPF and non-IPF cases in the training (${\mathrm{CCR}}^{\mathrm{Tr}}$) and test (${\mathrm{CCR}}^{T}$) of the model. Additionally, the CCR for non-IPF (${\mathrm{CCR}}_{C_{0}}^{\mathrm{Tr}}$ and ${\mathrm{CCR}}_{C_{0}}^{T}$) and IPF classes (${\mathrm{CCR}}_{C_{1}}^{\mathrm{Tr}}$ and ${\mathrm{CCR}}_{C_{1}}^{T}$) are considered. This metric is computed from a contingency or confusion matrix (CM), which compares actual and predicted classifications and computes the number of true positives, true negatives, false positives, and false negatives. Thus, the CMs for training and test are evaluated (${\mathrm{CM}}^{\mathrm{Tr}}$ and ${\mathrm{CM}}^{T}$) for the global LMM model.

## 3 Results

The purpose of this section is two-fold. Firstly, we aim to empirically test the goodness of fit of the proposed method when compared to established state-of-the-art models (Section 3.1). This will involve a thorough examination of the performance metrics of each model and a comparison of their predictive accuracy. Secondly, we will conduct a detailed analysis of the best performing model in terms of its performance and its parameters (Section 3.2).

### 3.1 Computational experiments

In this study, we compare the performance of different state-of-the-art computational models for the detection of IPF and non-IPF with the LMM model. This analysis aims to provide evidence that the LMM model is a competitive and adequate approach for IPF case detection with the stratification of coping strategies. We employ various methods, including Bayesian networks (BayesNet and NaivesBayes), decision trees (RandomTree, RandomForest, and C4.05), lazy learning (K-Nearest Neighbor with Euclidean and Entropy distance functions), and function-based learning (Radial Basis Functions Neural Networks and Logistic regression with and without regularization). These diverse methods allow for a comprehensive evaluation of the LMM model's performance compared to other models in the literature.

Taking into account the different nature of the methods, they were compared with metrics derived from the confusion matrix such as general CCR (${\mathrm{CCR}}^{T}$) and the CCR by class (${\mathrm{CCR}}_{C_{0}}^{T}$ and ${\mathrm{CCR}}_{C_{1}}^{T}$) (in the test set) as they are common to all classification methods. To ensure the reliability of our results, we employed a hold-out cross-validation method, repeated 30 times. The dataset was randomly partitioned into training and test sets using a randomized seed, with the training set containing 66% of cases and the test set containing the remaining 34%. This allowed us to evaluate the performance of our models on different sets of data, ensuring that our results are representative and not influenced by the particular data split used. As a result, there were 30 random datasets with different cases in training and test sets, reporting each of these 30 datasets a specific ${\mathrm{CCR}}_{C_{0}}^{T}$, ${\mathrm{CCR}}_{C_{1}}^{T}$, and ${\mathrm{CCR}}^{T}$. The Table 2 shows the mean and standard deviation of these measures for each method analyzed.

**Table 2:** Mean and standard deviation of the measures obtained from the different methods analyzed.

Algorithms	Performance metrics (Test set)		
	${\mathrm{CCR}}_{C_{0}}^{T}$	${\mathrm{CCR}}_{C_{1}}^{T}$	CCR^T^
BayesNet	84.01_3.10_	69.90_6.18_	78.39_2.65_
NaivesBayes	79.13_7.32_	66.06_18.33_	72.85_4.60_
RandomTree	81.66_2.79_	62.04_7.96_	73.23_3.27_
RandomForest	**88.28**_**2.90**_	63.01_7.00_	80.04_2.82_
C4.05	81.16_4.03_	60.70_7.70_	73.13_3.05_
K-Nearest Neighbor with Euclidean distance function	80.81_2.59_	68.50_5.63_	75.45_1.86_
K-Nearest Neighbor with Entropy distance function	82.10_2.90_	60.16_7.91_	75.25_2.83_
Radial Basis Function Neural Network	85.96_3.20_	64.67_7.96_	77.33_2.68_
Logistic regression with regularization	85.97_4.05_	67.88_7.24_	77.05_2.64_
Logistic regression without regularization	82.03_3.74_	68.85_7.10_	76.39_3.07_
LMM model	79.51_0.87_	**77.03**_**0.45**_	**80.10**_**0.97**_

As can be seen in Table 2, the proposed models correctly detect a high percentage of IPF and non-IPF cases. The LMM model has the best ${\mathrm{CCR}}_{C_{1}}^{T}$, and ${\mathrm{CCR}}^{T}$ results (the critical elements in the research study). The classification model proposed in this study is particularly competitive when predicting the $C_{1}$ class, which corresponds to IPF. This class is of special interest in the study, making the proposed model a significant contribution to the field. The results of the study indicate that the second-best model for IPF case detection is Random Forest. It is worth noting that Random Forest is an ensemble method that combines multiple decision trees to improve the model's predictive power. However, due to its complex internal workings, it can be challenging to understand how the model arrives at its predictions. This complexity may pose a challenge when interpreting the results and discussing the model's performance in the discussion section. Therefore, it is important to consider not only the model's accuracy but also its interpretability when selecting the most suitable method for a given task.

The model proposed has two distinctive characteristics that make it perform better in IPF and non-IPF detection than the others analyzed. First, it integrates linear relationships but also nonlinear relationships of the 2×2 interactions of independent variables on IPF. Therefore, the LMM model identifies the direct effect of specific independent variables on IPF, but also detects changes in that effect considering the interaction between them. Second, the LMM model considers the stratified nature of the IPF phenomenon, which is not possible through other models. Accordingly, the LMM model is the most appropriate model to address the objectives of the study. For this reason, the following section shows a LMM model with the best performance in IPF detection out of the 30 obtained in the hold-out.

### 3.2 Analysis of the best performing model

Table 3 presents the best-performing LMM model obtained during optimization. The model includes a fixed effects model with common predictors for IPF between individuals, irrespective of whether they utilize engagement or disengagement coping strategies. Additionally, the LMM model incorporates two random effects models ($G_{1}$ and $G_{2}$) that augment the baseline model. Specifically, the $G_{1}$ model includes unique predictors for individuals using disengagement coping strategies, while the $G_{2}$ model has distinct predictors for individuals using engagement coping strategies.

**Table 3:** LMM model obtained during the optimization process (SE: Standard error of the coefficients, CM^Tr^,CM^T^) confusion matrices for the training and test sets, CCR^Tr^,CCR^T^ Correct Classification Rate (CCR) for the training and the test sets, ${\mathrm{CCR}}_{C_{0}}^{\mathrm{Tr}},{\mathrm{CCR}}_{C_{1}}^{\mathrm{Tr}},{\mathrm{CCR}}_{C_{0}}^{T},{\mathrm{CCR}}_{C_{1}}^{T}$ CCR for the training and test sets for the two classes considered, $C_{0}$ and $C_{1}$.

Statistical properties of the LMM model			
	Estimate	SE	t-stat

**Fixed Effects**			
Frequency of violence (*x*_31_)	0.852	0.329	2.591
Frequency of violence (*x*_31_) x escalation of violence (*x*_32_)	0.481	0.182	2.644
Frequency of violence (*x*_31_) x community presence (*x*_38_)	-0.395	0.094	-4.210
Physical violence (*x*_33_) x situational characteristic of crime (*x*_37_)	0.144	0.061	2.372
Situational characteristic of crime (*x*_37_) x history of injuries (*x*_22_)	-0.230	0.116	-1.987
*Statistics:*			
Root Mean Squared Error: 0.323			
R-squared: 0.425			

**Random effects model**$G_{1}$**(Disengagement coping strategies)**			
Intercept	0.568	0.198	2.866
History of sexual aggression (*x*_27_)	-0.858	0.334	-2.570
*Statistics:*			
Root Mean Squared Error: 0.247			
R-squared: 0.683			

**Random effects model**$G_{2}$**(Engagement coping strategies)**			
Intercept	-0.275	0.095	-2.884
Frequency of violence (*x*_31_) x escalation of violence (*x*_32_)	-0.625	0.293	-2.136
Frequency of violence (*x*_31_) x physical violence (*x*_33_)	0.945	0.346	2.729
Physical violence (*x*_33_) x place of crime (*x*_35_)	-0.766	0.311	-2.462
Place of crime (*x*_35_) x community presence (*x*_38_)	0.884	0.342	2.587
*Statistics:*			
Root Mean Squared Error: 0.297			
R-squared: 0.530			

Classification performance of the LMM model			
${\mathrm{CM}}^{\mathrm{Tr}}=\left(\begin{array}{cc} 208 & 13\\ 27 & 80 \end{array}\right)$; ${\mathrm{CM}}^{T}=\left(\begin{array}{cc} 90 & 19\\ 12 & 42 \end{array}\right)$			
${\mathrm{CCR}}^{\mathrm{Tr}}=0.878$; ${\mathrm{CCR}}^{T}=0.810$			
${\mathrm{CCR}}_{C_{0}}^{\mathrm{Tr}}=0.941$; ${\mathrm{CCR}}_{C_{0}}^{T}=0.825;{\mathrm{CCR}}_{C_{1}}^{\mathrm{Tr}}=0.758$; ${\mathrm{CCR}}_{C_{1}}^{T}=0.777$			

The LMM model's classification performance exhibited a training CCR of 0.878 (${\mathrm{CCR}}^{\mathrm{Tr}}$) and a test CCR of 0.810 (${\mathrm{CCR}}^{T}$). These results were derived from the confusion matrices of the training (${\mathrm{CM}}^{\mathrm{Tr}}$) and test (${\mathrm{CM}}^{T}$) phases, respectively, which included 208 true positives, 13 false negatives, 27 false positives, and 80 true negatives in the training set, and 90 true positives, 19 false negatives, 12 false positives, and 42 true negatives in the test set. The CCR for non-IPF cases in the training phase (${\mathrm{CCR}}_{C_{0}}^{\mathrm{Tr}}$) was 0.941, while in the test phase (${\mathrm{CCR}}_{C_{0}}^{T}$), it was 0.825. Conversely, the CCR for IPF cases in the training phase (${\mathrm{CCR}}_{C_{1}}^{\mathrm{Tr}}$) was 0.758, whereas in the test phase (${\mathrm{CCR}}_{C_{1}}^{T}$), it was 0.777.

As can be seen, the models proposed yield R^2^ values of 0.42, 0.68, and 0.53, respectively. In line with the reference [53], it is well-established that R^2^ values falling between 0.10 and 0.50 are generally considered acceptable in social sciences, provided that a significant proportion of the variables are statistically significant. In this study, two of the three models implemented surpass the 0.50 threshold, and notably, each of their constituent variables demonstrates statistical significance.

The next subsections will describe the fixed effects and random effects models in detail. These models are essential components of our analysis and provide valuable insights into the relationship between IPF and coping strategies. The fixed effects model consists of a common set of predictors that are applied to all individuals, while the random effects models include unique predictors for different subgroups. By explaining the specific features of these models, we aim to enhance the reader's understanding of the underlying dynamics and mechanisms of the IPF-coping strategies relationship.

#### 3.2.1 Fixed effects model

The fixed effects model, $f^{\mathrm{FE}}$, is mathematically defined as:

$$f^{\mathrm{FE}}(x)=0.852x_{31}+0.481x_{31}x_{32}-0.395x_{31}x_{38}+0.144x_{33}x_{37}-0.230x_{37}x_{22},$$

where **x** represents the vector of predictor variables. As can be seen, the estimated coefficient for the frequency of violence variable ($x_{31}$) is 0.852, indicating a strong positive relationship between this variable and IPF. This means that as the frequency of violence increases, so does the probability of experiencing IPF. When the frequency of violence variable is combined with the escalation of violence variable ($x_{32}$), the estimated coefficient is 0.481, indicating a positive effect on IPF again. However, we also observe negative effects when the frequency of violence variable is combined with the community presence variable ($x_{38}$). In this case, the estimated coefficient is -0.395, implying a moderate decrease in the likelihood of IPF occurrence.

The interaction between the physical violence ($x_{33}$) and situational characteristic of crime ($x_{37}$) variables has a low positive effect on IPF, as indicated by the estimated coefficient of 0.144. When the values of both variables increase, the probability of IPF occurrence slightly increases. On the other hand, the combination of the characteristics of crime ($x_{37}$) and history of injuries ($x_{22}$) variables has a negative effect on IPF, with an estimated coefficient of -0.230. As the values of these variables increase, the probability of IPF occurrence decreases slightly.

The goodness-of-fit analysis indicates a highly precise and reliable fit of the fixed effects model to the dataset, as supported by both the RMSE and R^2^ values. The low RMSE value of 0.323 indicates that the model has a small mean difference between the actual and predicted values, implying that the model is accurate in predicting the dependent variable. Moreover, the R^2^ value of 0.425 shows that the model can explain 42.5% of the variance in the dependent variable using the predictor variables included in the model. These results demonstrate that the fixed effects model is a suitable and effective approach for explaining the relationship between the predictor variables and the dependent variable.

#### 3.2.2 Random effects model $G_{1}$ (disengagement coping strategies)

The random effects model $G_{1}$, $f^{\mathrm{RE}(G_{1})}$, is an extension of the fixed effects model that includes an additional intercept and a variable specific to cases that utilize disengagement coping strategies. Therefore, the equation for model $G_{1}$ is represented as follows:

$$f^{\mathrm{RE}(G_{1})}(x)=f^{\mathrm{FE}}(x)+0.568-0.858x_{27}.$$

As can be seen in the equation, the intercept in the model has a positive effect on IPF, with a value of 0.568. This suggests that victims with disengagement coping strategies are more likely to experience IPF. Furthermore, the model includes an additional predictor variable, history of sexual aggression ($x_{27}$), which negatively and intensely affects IPF. The estimated coefficient for $x_{27}$ is -0.858, indicating that a history of sexual aggression almost completely nullifies the risk of IPF. These results highlight the importance of considering individual differences in coping strategies and previous experiences when examining the risk factors for IPF.

The performance of model $G_{1}$ on the disengagement coping strategies dataset is highly satisfactory, as indicated by the RMSE and R^2^ results. The low RMSE value of 0.247 suggests a reduced difference between the actual and predicted values, indicating that the model is highly accurate. Additionally, the high R^2^ value of 0.683 indicates that the model can explain 68.3% of the variance in the dependent variable using the predictor variables included in the model. Overall, these results demonstrate that model $G_{1}$ is highly effective in explaining the relationship between the predictor and dependent variables in cases with disengagement coping strategies.

#### 3.2.3 Random effects model $G_{2}$ (engagement coping strategies)

The random effects model $G_{2}$, $f^{\mathrm{RE}(G_{2})}$, is a combination of the fixed effects model with an additional intercept and several pairs of variables that apply only to cases with engagement coping strategies. The equation of the model $G_{2}$ can be expressed as follows:

$$f^{\mathrm{RE}(G_{2})}(x)=f^{\mathrm{FE}}(x)-0.275-0.625x_{31}x_{32}+0.945x_{31}x_{33}-0.766x_{33}x_{35}+0.884x_{35}x_{38}.$$

As can be seen in the equation, the intercept has a negative and low effect on IPF (value -0.275). This means that victims with engagement coping have a slight decrease in the probability of suffering IPF. These victims are a priori less predisposed to suffer IPF. Another particularity of the model $G_{2}$ is that it adjusts the estimated coefficient of the combination of the frequency of violence ($x_{31}$) and the escalation of violence ($x_{32}$) variables from the fixed effects model to cases with engagement coping strategies. This coefficient is -0.625, and it involves that the interaction of both variables has a negative and moderate effect on IPF.^12^ The greater the increase of the frequency of violence ($x_{31}$) and the escalation of violence ($x_{32}$), the less the probability of occurrence of IPF and vice-versa.

The first additional interaction of variables of the model $G_{2}$ refers to the frequency of violence ($x_{31}$) and the physical violence ($x_{33}$). Its estimated coefficient is 0.945, which means that both variables are positively and intensely related to IPF. The greater the increase of values in the frequency of violence ($x_{31}$) and the physical violence ($x_{33}$) variables, the greater the probability of occurrence of IPF. The second additional interaction of variables concerns the physical violence ($x_{33}$) and the place of the crime ($x_{35}$). Both variables negatively and significantly affect IPF since the estimated coefficient is -0.7666. The probability of IPF occurrence decreases as the value of physical violence ($x_{33}$) and place of the crime ($x_{35}$) variables increases, and vice-versa. The third additional combination of variables consists of the place of the crime ($x_{35}$) and community presence ($x_{38}$). It positively and strongly affects IPF because its estimated coefficient is 0.884. The probability of IPF occurrence increases as the values of the place of the crime ($x_{35}$) and the community presence ($x_{38}$) increase.

Regarding the performance of model $G_{2}$, the RMSE and R^2^ results demonstrate that this model provides a competitive fit to the engagement coping strategies dataset. Specifically, the low RMSE value of 0.297 indicates a small mean difference between the predicted and actual values. Additionally, the R^2^ value of 0.530 indicates that the model explains 53% of the variance in the dependent variable using the predictor variables included in the model. These results highlight the effectiveness of the model $G_{2}$ in explaining the relationship between the predictor and dependent variables in the engagement coping strategies dataset.

## 4 Discussion

The study responds to the proposed objectives by developing the dynamic multilevel linear mixed model (LMM) with common and specific risk indicators to engagement and disengagement coping strategies. The model obtained shows competitive performance in estimating IPF. Its correct classification rate is high, the R-squared value is above the accepted 0.10 in social sciences, and the mean square error is low. This study utilizes official data from the Vlex database, which ensures the reliability of the results obtained. This database provided information on the coping strategies used by the women who were murdered, information that unfortunately cannot be obtained through interviews with them. That is, the mentioned database provides us knowledge of the strategies used by women who have suffered the most severe violence (which, as the results of the study show, can be either engagement or disengagement coping strategies), making this study particularly valuable in contributing to the literature on IPF.

The model has detected eight risk indicators significantly associated with IPF, both individually and in second order interactions: $x_{22}$, $x_{27}$, $x_{31}$, $x_{32}$, $x_{33}$, $x_{35}$, $x_{37}$, and $x_{38}$. In certain scenarios, the same independent variable can have different effects on IPF depending on its interaction with other independent variables. These interactions suggest that a third independent variable moderates the relationship between the independent and dependent variables. For instance, the frequency of violence ($x_{31}$) has been shown to increase the likelihood of IPF, in line with prior research [14], [54], [55], [56], [57]. However, when the frequency of violence ($x_{31}$) is combined with community presence ($x_{38}$), the probability of IPF actually decreases. Community presence acts as a buffer to the frequency of violence variable, protecting the victim from IPF when violence is present. Prior studies have suggested that the presence of people during violent incidents could deter the aggressor from producing IPF, indicating that community presence has a negative effect on IPV [58], [59], [60]. Conversely, a lack of community could increase the likelihood of violence by reducing the perceived costs of committing a crime, such as sanctions [61], [62]. Our study is the first to analyze the role of community presence in the context of IPF, offering novel insights into IPF.

The same principle applies to the situational characteristic of the crime variable ($x_{37}$), as its effects on IPF depend on its interaction with other variables. When combined with physical violence ($x_{33}$), the probability of IPF increases. When combined with the variable history of injuries ($x_{22}$), the probability decreases. Previous studies reveal that both physical violence ($x_{33}$) and history of criminal acts on which history of injuries ($x_{22}$) are considered factors associated with IPF [63], [64], [65], [55], [66], so the variable situational characteristic of crime ($x_{37}$) could explain why there is an increase and a decrease in the IPF probability in the mentioned interactions.

Environmental criminology theories suggest that criminal behavior is not solely the result of an individual's propensity for committing crimes, but it is also influenced by the environment in which the crime occurs. The characteristics of the environment can affect a criminal's decision-making process and behavior, either encouraging or inhibiting the commission of crimes [67], [68], [69], [70], [71], [72]. Solitary locations are generally considered criminogenic, as they provide an opportunity for offenders to commit crimes with little risk of being caught. This is consistent with rational choice theory, which suggests that perpetrators weigh the costs and benefits of committing a crime [73], [74]. However, in the case of IPF, lonely places are not criminogenic when it is associated to history of injuries. According to Cohen and Felson [68], crime is committed in a specific location with a motivated aggressor, an accessible victim and a lack of adequate surveillance. This could explain why given the opportunity to be in a solitary place with a victim in which there is no surveillance to prevent the commission of IPF, aggressors motivation is crucial. Only aggressors with motivation to kill women commit IPF, and not those without it [70].

To better understand the counter-intuitive negative effect of the interaction between the situational characteristic of crime ($x_{37}$) and the history of injuries ($x_{22}$) on IPF, we conducted an analysis of cases with these variables (89 cases out of the total 491 in the database). We calculated the mean and median of the variables for these specific cases and obtained a prototype that refers to women who suffer repeated violence over time, resulting in injuries, and proactively cope with it. This prototype may explain the negative estimate obtained in the aforementioned interaction, where the victim's actions can prevent IPF. Additionally, we suggest that some aggressors may exert violence on the victim as a way of domination, control, and submission to maintain their male role in a patriarchal society, without a motivation for IPF. This could also explain why a solitary place, in this case, would not be criminogenic.

The variable of place ($x_{35}$) is another factor that can influence the likelihood of IPF for victims who use engagement coping strategies, and its effect depends on the other variables it combines with. When victims with engagement coping strategies experience physical violence ($x_{33}$) in urban areas ($x_{35}$), the probability of IPF is reduced. This finding aligns with previous research suggesting that women from urban areas have better access to victim assistance services, and thus are more likely to seek help than their rural counterparts [25]. Conversely, the probability of IPF increases when victims with engagement coping strategies experience violence in urban areas ($x_{35}$) with high levels of community presence ($x_{38}$). The strength of social ties between community members is a critical factor in maintaining community presence, and weakened ties can result in increased levels of crime in the area [18], [19]. In rural areas the disposition of neighbors to help and the perception that they are close and trustworthy are significantly higher. Building on these findings, we hypothesize that the anonymity that is characteristic of urban areas may contribute to individuals failing to act as community presence in preventing IPF, including failing to alert the police. This could explain the increased likelihood of IPF occurring when urban areas are combined with a lack of community.

The result regarding the variable of history of sexual aggression for disengagement coping is contradictory to previous studies [75], [76], as it was found that having a history of violence decreases the probability of IPF, while previous studies indicate the opposite [75], [64], [77], [78]. To better understand this result, an additional analysis was conducted on the cases with a history of sexual aggression included in the database, which amounted to only 8 cases out of the 491. Of these 8 cases, 2 were classified as IPF. Given the small number of cases, the result obtained regarding the history of sexual aggression must be interpreted with caution.

The study has also found evidence that the same interaction between variables has a different effect on IPF in cases with disengagement and engagement coping strategies. The fixed-effects model reveals that when the frequency of violence ($x_{31}$) is combined with the escalation of violence ($x_{32}$), the probability of IPF increases [79], [56]. However, this only applies to cases with disengagement coping strategies, as the random-effects model reveals that this interaction decreases the probability of IPF in cases with engagement coping strategies. These findings suggest a protective effect of engagement strategies on IPF under these conditions. It is hypothesized that engagement strategies can stop the cycle of violence inherent to IPV. When this cycle is repeated, the frequency and severity of violence escalate, leading to IPF [80]. This study reveals that this only applies to cases with disengagement coping strategies, not engagement cases. However, further research is necessary to confirm this hypothesis.

Concerning those mentioned above, several studies indicate that victims exposed to severe and recurrent violence face significant obstacles in developing effective coping strategies (such as seeking professional support, reporting the abuse, or ending the relationship). This fact hinders the cessation of the cycle of violence, leaving women more susceptible to further violence [81], [82], [83], [84]. Among the scientific-evidenced obstacles are the victim's fear of retaliation by the aggressor, economic dependence on the aggressor, social isolation, and feelings of shame and guilt in the victim. The victim's fear of retaliation by the aggressor commonly manifests in threats of additional violence or even death if the victim takes action against violence perpetrated by their (ex)partners. Economic dependence on the abuser can also generate apprehension about the possibility of losing their source of income if they decide to take action. Social isolation represents another critical obstacle, as isolated victims experience greater difficulty confiding in friends and family to tell about the abuse, and isolation limits their access to resources for help and protection. In addition, the feelings of shame and guilt that some victims experience because of suffering violence, as well as the hope that the abuser will change, are emotions that could dissuade them from seeking assistance [85], [86], [87], [37]. For these reasons, it is essential to provide institutional support and resources that address these barriers and thus help the victims take action to safeguard their security and well-being.

According to some studies, the use of disengagement and engagement coping strategies is dependent on cognitive processes that involve recognizing violence, deciding and preparing to seek help, and taking action [88], [24]. Victims at the pre-contemplative stage may not be aware of the abuse, justifying the violence and minimizing its consequences. Victims at this stage may not seek help, so when they experience high-frequency violence accompanied by an escalation of frequency and severity of violence, the likelihood of IPF increases. At the contemplative stage, victims recognize the abuse and consider possible actions and resources to stop the violence while assessing the pros and cons of taking these actions. At this point, some victims may decide not to proceed to take action if they perceive that their cons outweigh their benefits. In contrast, victims who consider that taking action has more advantages than disadvantages pass to the preparation and action stages, seek help, and use engagement coping strategies. In these cases, high-frequency violence accompanied by an escalation of frequency and severity of violence could not increase the likelihood of IPF because the victims take action to prevent it [88], [24]. These assumptions could also explain why victims with disengagement coping strategies have a higher predisposition to suffer IPF than victims with engagement coping strategies as revealed by the study's results. At this point, it is important to recognize that some women, despite being aware of the abuse and their desire to stop it, do not have alternatives to escape the violence for a variety of barriers such as economic and/or emotional dependence to aggressor, lack of social support or fear of death threats becoming a reality.

As the findings reveal that disengagement coping is a significant predictor of IPF, risk assessment instruments should take this into account. IPF prevention programs should prioritize addressing these modifiable coping strategies factors. Awareness-raising programs are necessary to help women detect signs of lethal abuse and become aware of their situation, thereby increasing their willingness to take action. Empowerment interventions, including training in engagement strategies, are also crucial to provide concrete and effective action guidelines. Professional services are unable to assist victims who do not seek help; thus, empowering women who suffer from violence to report the abuse is not enough. Many victims are unaware that they are victims, and others, despite being aware, do not know how to deal with it. Situational crime prevention could also be effective in IPF cases by reducing the conditions that make solitary places ideal for IPF crimes, such as enhancing street lighting, incorporating video surveillance services, and strengthening community links that contribute to community presence of crime and social support [89], [90]. In addition to prevention programs, accessible services with sufficient and effective resources are essential to assist victims, especially in rural areas, as evidenced by the findings with regard to location.

To effectively address violence against women by intimate partners, governments must provide comprehensive support to victims, including material, economical, psychological, social, and legal resources. Concerning material support, it is essential to expand professional services to residential areas where the existing may be far from the reach of those in need. Bringing these services closer to women experiencing violence makes it easier for them to seek assistance and support. Additionally, more resources should be allocated for accessible housing for victims who need a safe place to stay, and thus, ensure that the fact that victims have nowhere to live is not an obstacle to taking engagement coping strategies. Regarding economic support, the government must provide financial aid as well as training and employment programs for victims who lack education and employment and are economically dependent on their abusers. This support could reduce barriers for victims to develop engagement coping strategies.

Equally important is the allocation of resources by the government to victim support services and organizations to effectively address intimate partner violence. These services are indispensable for providing psychological support and therapy to help victims cope with the abuse trauma. Additionally, these organizations commonly provide victims with legal advice, ensuring that the lack of information about reporting and legal proceedings is not a hindrance to taking action. These services and organizations facilitate establishing support networks among victims, essential for expanding victims' social circles, particularly when they may be socially isolated.

The current study focuses on the coping strategies of victims, emphasizing the importance of interventions aimed at victims in terms of prevention. However, interventions focused only on victims taking action to prevent IPF are insufficient. Controlling the violent behaviors of aggressors is crucial to prevent them from victimizing women. Therefore, implementing specific programs directed at the aggressors, including education, treatment, and guidance to modify their behavior, becomes a fundamental element for effectively preventing IPF.

The present study has several limitations that should be acknowledged. First, the study was conducted only in Spain, and therefore, the generalizability of the findings to other countries is uncertain. Second, the study's time frame is limited to cases reported between 2019 and 2022, and hence the findings may not be applicable to cases reported in other years. Third, the study only includes cases involving adult victims and offenders, and therefore, the results may not be generalizable to cases involving children. Fourth, the retrospective nature of the study means that no follow-up of the cases has been conducted, and it is unclear whether any IPV cases have escalated to IPF. Fifth, the restricted sample size of cases with a history of sexual aggression introduces inherent limitations regarding the robustness and generalizability of the study's findings. The small number of cases makes it impossible to draw inferences regarding the impact of this variable on the IPF. Finally, the study relies exclusively on legal documents for data collection, and therefore, any information not included in these documents has not been considered. Accordingly, the current research has not been able to assess information related to psychological and interpersonal aspects, as such data was not available in the documents considered for this investigation. Despite these limitations, the findings of this study provide valuable insights into the risk factors associated with IPF and the importance of coping strategies in reducing this risk.

## 5 Conclusions

The study makes a significant contribution to advancing our understanding of Intimate Partner Violence (IPF), by examining coping strategies and analyzing data gathered from justice professionals. The multilevel linear mixed model employed in the study demonstrated a competitive performance in distinguishing between IPF and non-IPF incidents, utilizing a range of risk indicators including prior history of injuries, history of sexual aggression, frequency and escalation of violence, physical violence, location of crime, and community presence. While some of these factors were found to be common to both coping groups, others were found to be characteristic of one group or the other. Importantly, the study also revealed that victims who utilize disengagement coping strategies are more likely to suffer from IPF than those who use engagement coping strategies. This evidence-based knowledge underscores the urgent need for effective interventions and support services to promote engagement coping strategies in victims and reduce their risk of harm.

## CRediT authorship contribution statement

**Esperanza Garcia-Vergara:** Writing – review & editing, Writing – original draft, Project administration, Methodology, Funding acquisition, Formal analysis, Conceptualization. **Francisco Fernández-Navarro:** Writing – review & editing, Validation, Supervision, Software, Resources, Project administration, Methodology, Formal analysis, Conceptualization. **David Becerra-Alonso:** Supervision, Software, Methodology, Formal analysis, Conceptualization. **Nerea Almeda:** Validation, Supervision, Investigation, Conceptualization.

## Declaration of Competing Interest

The authors declare that they have no known competing financial interests or personal relationships that could have appeared to influence the work reported in this paper.

## Data Availability

The data that support the findings of this study, as well as the codes used for analysis, are available from the corresponding author upon request.

## Footnotes

2 Injuries of IPV crime refers to acts in which a man harms the physical and/or psychological health of his female (ex-)partner.

3 Threats of IPV crime involves manifestations of harm by a man to his female (ex-)partner.

4 Constrains of IPV crime entails the use of violence by a male, without being legitimately authorized, to impede his female (ex-)partner from doing something or to force her to do something against her will.

5 Habitual violence of IPV crime implies the habitual exercise of physical or psychological violence by man against his female (ex-)partner with whom cohabits.

6 Insults of IPV crime refer to actions or expressions (oral or written) by a man that seriously injure the dignity of his female (ex-)partner.

8 Illegal detention of IPV crime refers to the deprivation of freedom of movement by a man to his (ex-) female partner, either by locking her up or detaining her.

9 Sexual aggression and abuse of IPV refers to assaults against sexual freedom of women by her male (ex-)partners, with the difference that sexual aggression is perpetrated with violence or intimidation and sexual abuse is not.

10 Invasion of privacy of IPV crime consists of illegal access and control of personal data of a woman by her male (ex-)partner.

## Footnotes

1 Vlex is an online platform that offers access to a large collection of up-to-date legal information from more than 150 countries worldwide. It contains a wide variety of law-related resources, including legislation and case law, among other legal documents. The platform provides users with advanced search tools and artificial intelligence filtering options, making locating specific legal information of interest easy. More information at https://vlex.cl.

7 Note that $x_{8}$ and $x_{9}$ are general variables that encompass all types of sentences. The rest of the variables provide additional information about specific types of sentences and their duration.

11 The model initially considers all the predictor variables and potential interactions among them, which leads to an optimization problem with a search space composed of 780 variables, 39 predictor variables (K=39), and 741 interactions terms (the binomial coefficient of 39 over 2).

12 The global estimate for the interaction $x_{31}$ and $x_{32}$ in the model $G_{2}$ is -0.144 since the estimation for the model $G_{2}$ is -0.625 and it has to consider the estimate of the fixed effects model that is 0.481.
